# *Mycobacterium tuberculosis* Rv0366c-Rv0367c encodes a non-canonical PezAT-like toxin-antitoxin pair

**DOI:** 10.1038/s41598-018-37473-y

**Published:** 2019-02-04

**Authors:** Himani Tandon, Arun Sharma, Sankaran Sandhya, Narayanaswamy Srinivasan, Ramandeep Singh

**Affiliations:** 1 Molecular Biophysics Unit, Indian Institute of Science, Bangalore, 560012 India; 2 Tuberculosis Research Laboratory, Vaccine and Infectious Disease Research Centre, Translational Health Science and Technology Institute, NCR Biotech Science Cluster, 3rd Milestone, PO Box #4, Faridabad, Haryana 121001 India

## Abstract

Toxin-antitoxin (TA) systems are ubiquitously existing addiction modules with essential roles in bacterial persistence and virulence. The genome of *Mycobacterium tuberculosis* encodes approximately 79 TA systems. Through computational and experimental investigations, we report for the first time that Rv0366c-Rv0367c is a non-canonical PezAT-like toxin-antitoxin system in *M. tuberculosis*. Homology searches with known PezT homologues revealed that residues implicated in nucleotide, antitoxin-binding and catalysis are conserved in Rv0366c. Unlike canonical PezA antitoxins, the N-terminal of Rv0367c is predicted to adopt the ribbon-helix-helix (RHH) motif for deoxyribonucleic acid (DNA) recognition. Further, the modelled complex predicts that the interactions between PezT and PezA involve conserved residues. We performed a large-scale search in sequences encoded in 101 mycobacterial and 4500 prokaryotic genomes and show that such an atypical PezAT organization is conserved in 20 other mycobacterial organisms and in families of class Actinobacteria. We also demonstrate that overexpression of Rv0366c induces bacteriostasis and this growth defect could be restored upon co-expression of cognate antitoxin, Rv0367c. Further, we also observed that inducible expression of Rv0366c in *Mycobacterium smegmatis* results in decreased cell-length and enhanced tolerance against a front-line tuberculosis (TB) drug, ethambutol. Taken together, we have identified and functionally characterized a novel non-canonical TA system from *M. tuberculosis*.

## Introduction

Bacterial toxin-antitoxin (TA) systems are plasmid or chromosome-encoded, mobile genetic elements expressed as part of the same operon^1–3^. Together with other mechanisms of stress tolerance like the SOS response or the second messenger guanosine pentaphosphate, TA systems have major contribution in bacterial persistence: a phenomenon where bacterial cells can transform into persister cells when under stress^4^. Under normal environmental conditions, antitoxins neutralize toxin activity by forming a tight TA complex. Under stress, antitoxins are proteolytically cleaved and the released toxin functions as an intracellular poison that acts on its cellular targets, which range from specific messenger ribonucleic acid (mRNA), transfer ribonucleic acid (tRNA), ribosomal ribonucleic acid (rRNA) to proteins like deoxyribonucleic acid (DNA) gyrase or elongation factor thermo-unstable (EF-Tu)^5–9^. In general, the toxins are always proteins whereas antitoxins can either be proteins or ribonucleic acids (RNAs)^8^.

Depending upon the nature of antitoxins, these systems can be categorised into six types from I to VI. Type I TA systems have antisense RNAs as antitoxins, which bind to the toxin mRNA whereas in type II TA systems, antitoxin is a protein moiety which directly interacts with the toxin^10,11^. Type III TA systems are composed of an RNA antitoxin which binds to its cognate toxin and supresses its activity^12^. Type IV TA systems also possess a protein antitoxin but unlike type II, toxin and antitoxin bind to a common target^13,14^. Antitoxins in type V TA systems are endoribonucleases, which specifically cleave toxin mRNA^15^. In type VI TA systems, the toxin and antitoxin are both proteins. Here, the antitoxin does not neutralize the toxin directly on binding but promotes its degradation by specific proteases^9,16^. Among these, type II TA systems are the most well characterized families of TA systems. Here usually, the antitoxins have two domains: an N-terminal DNA-binding domain which functions to regulate the TA operon expression and a C-terminal toxin-binding domain which inhibits the toxin from binding to its cellular targets^17^. In some systems, the DNA-binding function is carried out by a third protein in the operon^18,19^.

The TA systems are ubiquitously present in Gram-positive and Gram-negative bacteria, as well as in archaebacteria. Several studies have been carried out previously, to explore the distribution of these systems across bacterial and archaebacterial genomes^11,20,21^. Well-known examples of type II systems include RelBE, ParDE, HigBA, VapBC, MazEF, CcdBA, Phd-Doc and ε/ζ/ω^1^. Out of these, the VapBC, RelBE, HigBA, MazEF and ParDE systems are the most abundant. The genus *Mycobacterium* is interesting because it includes not only the pathogenic *Mycobacterium tuberculosis* complex (MTBC) members but also non-pathogenic bacteria such as *Mycobacterium smegmatis*. The reported number of TA loci varies tremendously among these species e.g. *M. tuberculosis* harbours 79 systems, *M. smegmatis* has 3 such systems and *Mycobacterium leprae* has no known report of any TA system^21,22^. Within mycobacteria, *M. tuberculosis* possesses representatives from all major classes of type II TAs in single or multiple copies *viz*. 50 VapBC, 10 MazEF, 3 HigBA, 2 ParDE, 3 RelBE and few other unclassified novel systems^1,23^. But thus far, there have been no reports on any homologues of either CcdBA, ε/ζ/ω, PezAT and Phd/Doc systems in *M. tuberculosis*.

The ε/ζ/ω system was first reported on the plasmid pSM19035 of *Streptococcus pyogenes*^24^. Since then there have been numerous reports showing the presence of this system in various Gram-positive and Gram-negative bacteria^11^. The chromosomal homologue of ε/ζ/ω, known as PezAT, was first reported in *Streptococcus pneumoniae*^25^. These two homologous TA systems show differences in their transcription regulation. An accessory protein called ω is required for the transcriptional regulation of ε/ζ/ω system whereas this function is carried out by the N-terminal region of PezA antitoxin, characteristic of type II TA systems^26^. Further, whereas the ω protein adopts the RHH motif^27^, the N-terminal of PezA adopts the helix-turn-helix (HTH) motif^25^. The C-terminal of PezA antitoxin shows a sequence identity of 21% with the ε antitoxin^25,28^. The toxin of both plasmid and chromosomal systems, namely the ζ and PezT toxins, share a common phosphotransferase fold and possess 42% sequence identity^25^. The cytotoxic effect of ζ/PezT toxin is conventionally exerted by phosphorylating the peptidoglycan precursor uridine diphosphate-N-acetylglucosamine (UNAG), utilising adenosine triphosphate (ATP), but more recently nicotinamide adenine dinucleotide (NAD) and uridine diphosphate-N-acetylmuramic acid (UNAM) have also been identified as substrates for these toxins^29,30^. In both PezAT and ε/ζ/ω systems, the antitoxin functions by binding to the P-loop motif present in the toxin, thus preventing binding of ATP^25,28^. The plasmid ε/ζ/ω system is responsible for post-segregational killing of the bacterial cell. In addition, PezAT has also been associated with virulence of *S. pneumoniae*^24,31^.

In the present study, we have performed rigorous sequence searches, using the predicted and characterised ε/ζ/ω and PezAT TA systems known to-date, as queries, to probe the *M. tuberculosis* genome for homologues. Our searches identified Rv0366c-Rv0367c as a homologue for the PezAT using both ε/ζ/ω and PezAT sequences from other microorganisms as queries. We demonstrate that inducible expression of PezT-like toxin of *M. tuberculosis*, Rv0366c inhibits growth of *Escherichia coli* and various mycobacterial species in a bacteriostatic manner. We were able to restore this growth defect by expressing Rv0366c along with the corresponding PezA-like antitoxin, Rv0367c. We also show that inducible expression of PezT-like toxin increases tolerance of *M. smegmatis* to ethambutol, a cell wall inhibitor. As per our knowledge, this is the first report on characterization of PezAT-like TA system in *M. tuberculosis* H_37_Rv. Future experiments would involve characterization of the functional target of PezT in *M. tuberculosis* and understanding its role in *M. tuberculosis* physiology and pathogenesis.

## Results

### Identification of a putative ζ/PezT-like toxin and PezA-like antitoxin in *M. tuberculosis* H_37_Rv

Homologues of the PezT and ζ toxin sequences from Pfam were identified in the *M. tuberculosis* genome using TBLASTN^32,33^. Of the 1054 query sequences, 337 identified the gene Rv0366c (O53701) as a homologue in *M. tuberculosis* H_37_Rv. The sequence identity of Rv0366c with its queries ranged from 30% to 80% with e-values between 1e^−10^ and 4e^−96^. Pfam domain assignments for Rv0366c also predict, with very high confidence (e-value of 2.1e^−13^), that this protein possesses the ζ toxin domain (PF06414). A detailed sequence comparison of Rv0366c with known ζ and PezT revealed that it lacks the N-terminal region, which in ζ and PezT adopts a helical structure (Figure S1). This region in ζ and PezT consists of a few antitoxin-binding residues. The remaining region of Rv0366c aligns well except for a short insertion in ζ and PezT at its C-terminal region.

**Figure 1:**
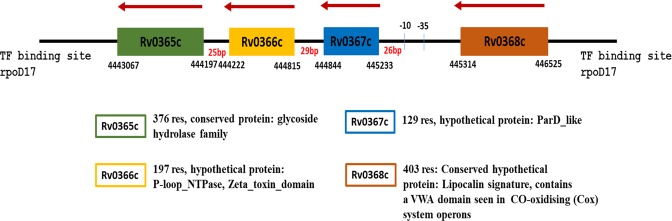
Representation of genomic region, ORF and promoters. The genomic locus for Rv0368c, Rv0367c, Rv0366c and Rv0365c are shown. Rv0367c, Rv0366c and Rv0365c were predicted to lie in the same operon. The promoter region was predicted to lie 26 bp upstream of Rv0367c^58^. The annotation for these gene neighbours, obtained from Tuberculist^59^ is also given below the figure. Rv0367c was considered as a putative antitoxin for further analysis.

After the assignment of potential ζ/PezT function to Rv0366c, we probed its neighbouring genes for a potential antitoxin. As shown in Fig. 1, Rv0365c, to the downstream of Rv0366c, is a 376-residue protein, which is annotated as a conserved glycoside hydrolase protein. To its upstream lies a 129 residue, hypothetical protein (Rv0367c), whose Pfam domain assignment suggested a ParD-like antitoxin (PF11903) domain for 65 out of 129 residues with an e-value of 4.7e^−22^. However, alignments with ParD-like antitoxin sequences showed poor conservation of toxin-binding residues (Figure S2). Secondary structure and disorder predictions of Rv0367c suggest that it is an ordered protein (data not shown), unlike the observed C-terminal of unstructured ParD antitoxin sequences^34^. Therefore, domain assignments of the Rv0367c to ParD antitoxins sequences were not considered for further evaluation.

### Rv0366c encodes a PezT/ ζ toxin homologue lacking a UNAG-binding site

**Figure 2:**
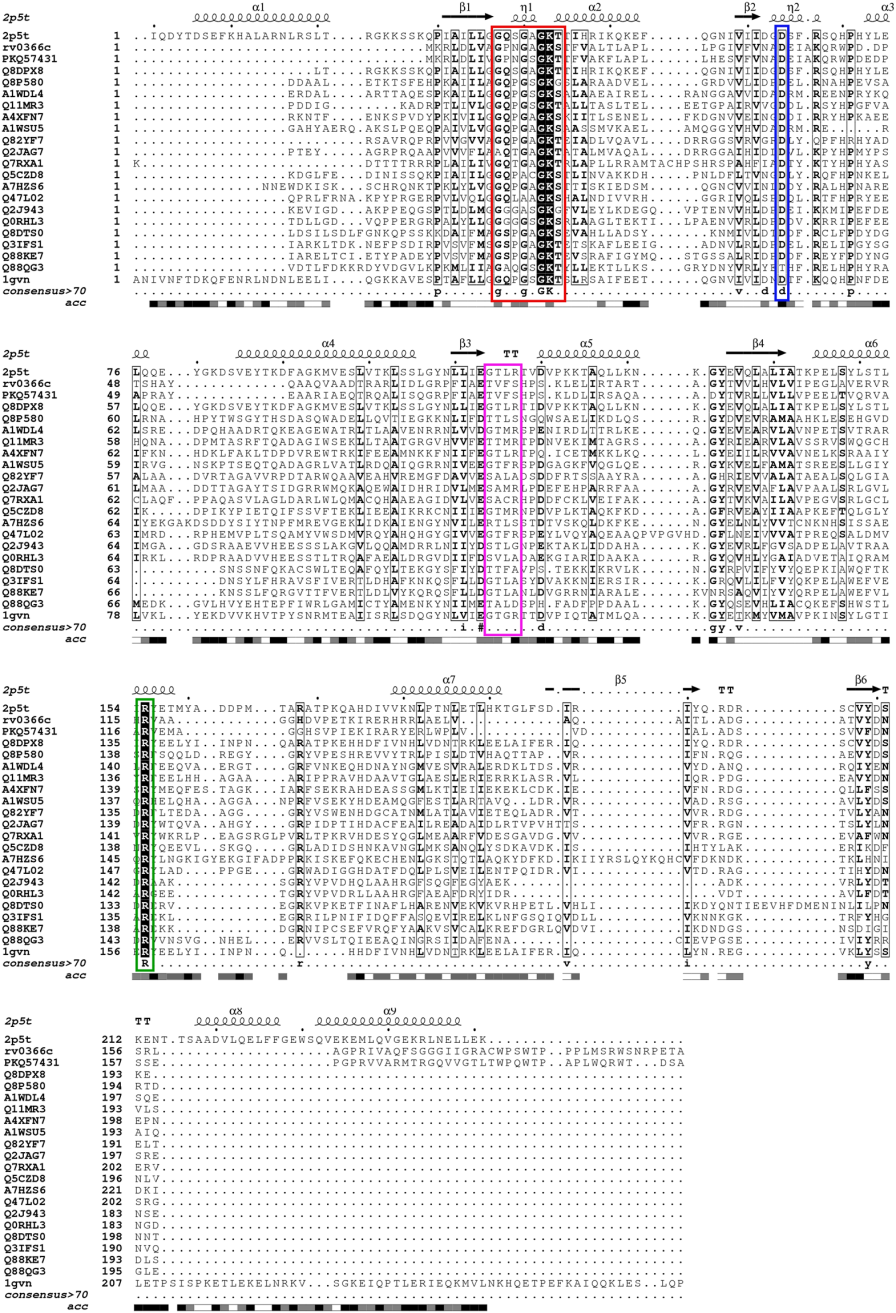
Structure-guided alignment of Rv0366c with ζ toxin from *Streptococcus pyogenes*, PezT from *Streptococcus pneumoniae* and other homologues. First sequence is the sequence of PezT of known structure. The nucleotide-binding motif is completely conserved and marked in red. The aspartate residue, important for deprotonation of substrate, is also conserved and marked in blue. The conserved arginine residue, marked in green, is important for both antitoxin-binding and catalysis. The other UNAG-binding residues are marked in pink. Other fully conserved residues are shown in black background, while semi-conserved residues in black boxes. Alignment was generated using ESpript 3.0^60^.

Rv0366c sequence was aligned with the sequences of known ζ toxin of *S. pyogenes* (1gvn) and PezT toxin of *S. pneumoniae* (2p5t) along with other members of the ζ toxin family from Pfam using MAFFT (Fig. 2)^25,28,35^. ζ/PezT toxins are UDP-N-acetylglucosamine kinases having a well-defined nucleotide-binding motif (GXXGXXKT)^25,28^. Apart from nucleotide-binding, the substrate-binding as well as the antitoxin-binding site are also conserved among the ζ/PezT toxins. The nucleotide-binding site in the ζ toxin structure (1gvn) includes residues harbouring the 40-GXXGXXKT-47 motif, Arg171 and Glu116. For PezT (2p5t), equivalent nucleotide-binding residues include 39-GXXGXXKT-46 motif, Arg170, Glu115^36^. As shown in Fig. 2, the nucleotide-binding residues are well-conserved in Rv0366c. These correspond to 9-GPNGAGKS-16, His122 and Glu75 in Rv0366c. An important antitoxin-binding residue is Arg158 in ζ toxin while in PezT, it corresponds to Arg157. This residue, in addition to its role in antitoxin binding, has also been shown to be important for enzyme catalysis. It was observed that this antitoxin-binding and substrate-binding residue is also well conserved in Rv0366c (Arg116, Fig. 2).

The GTXR motif lying between Gly117 to Thr121 in ζ and Gly116 to Thr120 in PezT toxins is required to bind UNAG^36^. In addition, Asp67, Glu100 in ζ and Asp66, Glu99 in PezT are part of the substrate-binding site. The residues Asp67 in ζ and Asp66 in PezT that lie in the nucleotide-binding site are important to deprotonate the substrate prior to phosphorylation and hence its activity^25,28,37^. The alignments in Fig. 2 show that the GTXR motif is not conserved in Rv0366c, but the Asp36 (equivalent to Asp67 and Asp66 in ζ and PezT respectively), is present in Rv0366c. The conservation of the nucleotide-binding residues and the residue required to prime the bound substrate for subsequent phosphorylation suggests that Rv0366c is also likely to function as a nucleotide kinase. The lack of conservation of the UNAG-binding residues suggests that it may target other cellular substrates. Since, Rv0366c is the chromosomal homolog of ζ and shares similarities with PezT, it makes a strong case for a non-canonical PezT toxin and would be referred as PezT^Mtb^ in subsequent experiments.

**Figure 3:**
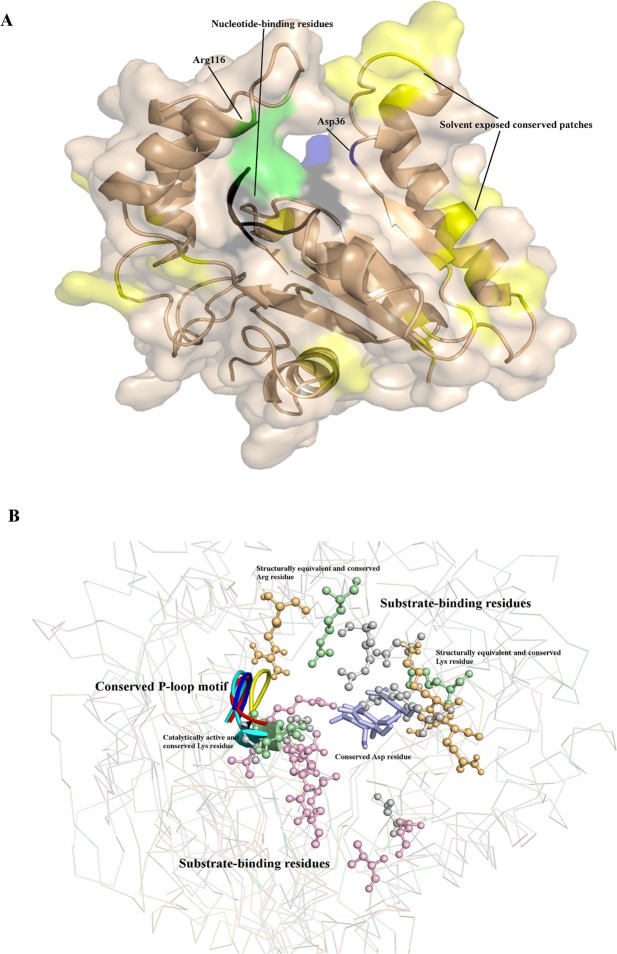
Modelled structure for PezT^Mtb^ and comparison of substrate-binding pockets of known ζ toxins with PezT^Mtb^. (**A**) Putative nucleotide-binding residues (black), conserved residue that may deprotonate the substrate (blue), residue with a potential role in both antitoxin-binding and catalysis (green) as well as solvent exposed, conserved residues, which include Lys40, Trp43, Pro44, His122, Val77 and Asn34 (yellow) are highlighted in the modelled PezT^Mtb^ structure. (**B**) Ribbon representation of superposed crystal structures of PezT (2p5t), ζ_ng (6epg), AvrRxo1 (4z8v) and the modelled structure of PezT^Mtb^. The nucleotide-binding P-loop motif is shown in cartoon representation, cyan- PezT, blue – ζ_ng, yellow- AvrRxo1 and red – PezT^Mtb^. The substrate-binding residues are shown in ball and stick, magenta- PezT, grey – ζ_ng, green - AvrRxo1 and orange– PezT^Mtb^. The conserved Asp residue, important for substrate-binding/deprotonating the substrate, is shown as sticks in light-blue. Other two residues (Lys40 and Arg116) known to bind substrate and are conserved in PezT^Mtb^ are also labelled.

In order to predict the three-dimensional organisation of the conserved residues and other surface exposed residues that may point to protein-protein interaction surfaces, PezT^Mtb^ was modelled using Modeller^38^. For this, 1gvn and 2p5t, which share 18% and 20% sequence identity respectively with Rv0366c, were used as templates^25,28^. Figure 3A shows the putative nucleotide-binding P-loop motif, Asp36 residue and the antitoxin-binding Arg116 residue. Other surface exposed and well conserved residues in accordance with the ζ/PezT toxin homologues (Lys40, Trp43, Pro44, His122, Val77 and Asn34) are also shown (Fig. 3A). These residues lie in the vicinity of the predicted ATP-binding motif and have been predicted to be solvent exposed in the modelled structure. Such conserved, solvent-exposed patches on protein structures can interact with substrate or antitoxin and hence are likely important for toxin function. This analysis raises the possibility that PezT^Mtb^ toxin can bind to a nucleotide and antitoxin despite being shorter than the canonical ζ/PezT toxins.

### Comparison of PezT^Mtb^ with ζ toxins that bind other substrates

Recently, it has been reported that ζ toxin in *Neisseria gonorrhoeae* (ζ_ng) is unable to phosphorylate UNAG at C3’-OH but phosphorylates UNAG, UNAM and other UDP-activated sugars at C4’-OH, hence showing broader and altered substrate specificity than the ζ and PezT from *S. pyogenes* and *S. pneumoniae* respectively^30^. Similarly, AvrRxo1 from *Xanthomonas Oryzae pv. oryzicola* is a ζ toxin family member that phosphorylates NAD in plants^29^. Hence, we compared these two ζ toxins with PezT^Mtb^. A structure-guided sequence alignment of PezT^Mtb^ with ζ_ng toxin is shown in Figure S3a and with AvrRxo1in Figure S3b. Figure S3a shows that PezT^Mtb^ which is shorter than ζ_ng (197, Rv0366c; 401, ζ_ng), lacks the substrate-binding (UNAM and UNAM-4P) residues seen in ζ_ng. Among the 10 substrate-binding residues identified in ζ_ng, only 3 (Asp36, Arg116 and His122) are conserved in PezT^Mtb^ (Table S1). For AvrRxo1, while precise information on the substrate-binding residues is not available, mutational experiments have identified two catalytic residues, Thr167 and Asp193 as important for phosphorylation of NAD^39^. Sequence alignment revealed that these two residues are conserved in PezT^Mtb^ (Ser16 and Asp36). Additionally, the predicted active-site residues for AvrRxo1, Lys15 and Arg116 are also conserved in PezT^Mtb^ (Table S1).

We further compared the structures of the canonical PezT (2p5t), ζ_ng (6epg) and AvrRxo1 (4z8v) and the modelled PezT^Mtb^ to analyse their ATP and substrate-binding pockets. Substrate-binding residues for the known structures along with predicted residues for AvrRxo1 were mapped on the available structures (Fig. 3B). We observed that while the ATP-binding P-loop motif is well conserved in all the structures, the substrate-binding cavity of PezT from *S. pneumoniae* differs from the other toxins. Between ζ_ng and AvrRxo1, PezT^Mtb^ showed better similarity to the substrate-binding region of AvrRxo1, both in terms of residue conservation and spatial location of the residues in the modelled structure (Figs 3B and S3). Hence, we speculate that the substrates for PezT^Mtb^ might be closer to AvrRxo1 substrates. Altogether, we predict that although the UNAG-binding residues are not conserved, the nucleotide-binding, the antitoxin-binding and the aspartate residue (Asp36), important for the activity of ζ/PezT toxins, are conserved, suggesting that Rv0366c might function as a PezT toxin but may target substrates other than UNAG.

### Rv0367c shows signatures of PezA/ε antitoxin revealing a non-canonical PezAT-like TA system

**Figure 4:**
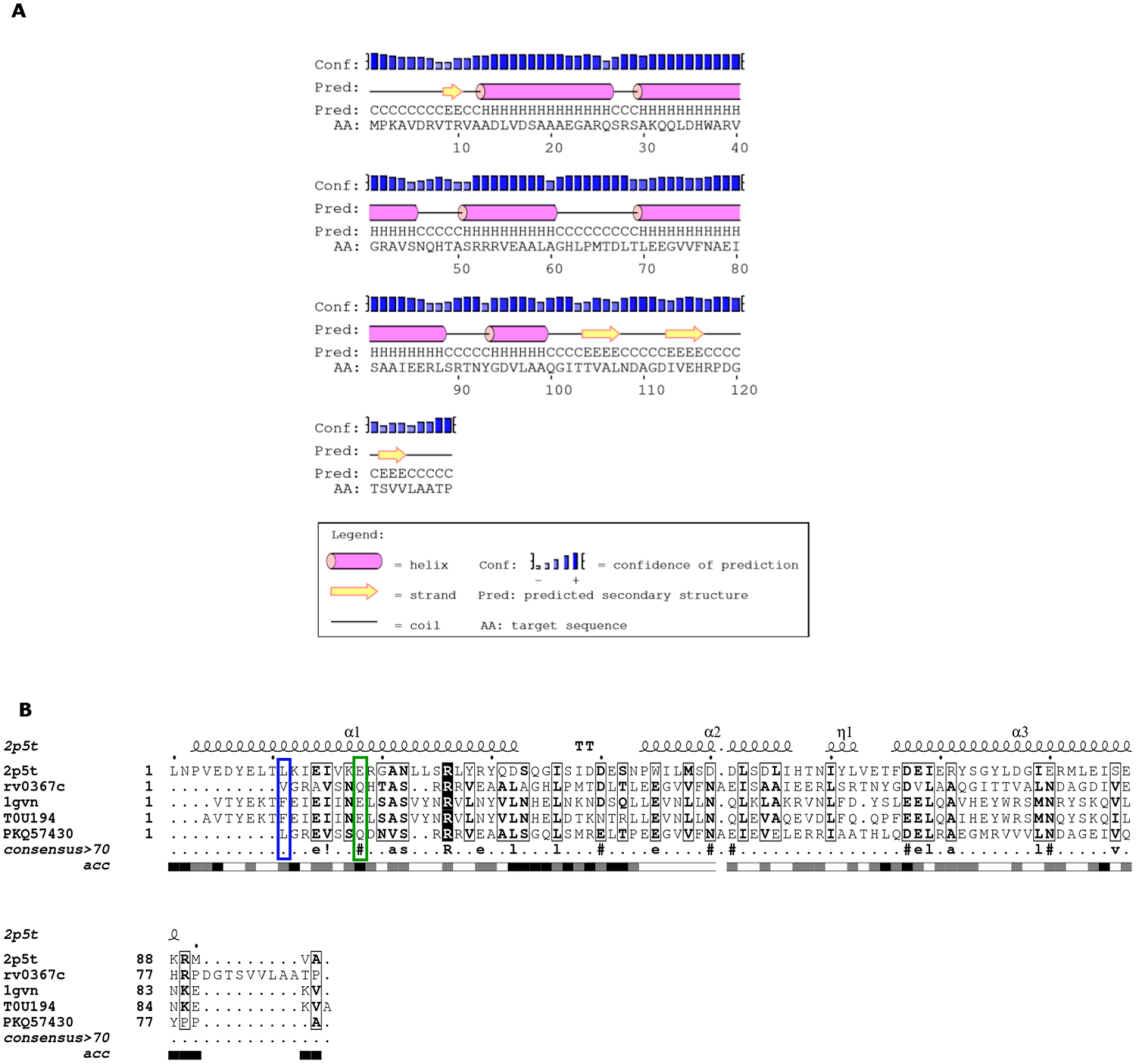
Alignment of PezA^Mtb^ with ε and PezA from other organisms. (**A**) The secondary structures predicted by PSIPRED show a ribbon-helix-helix (RHH) for the N-terminal region of Rv0367c^42^. C-terminal region is also predicted to be ordered with α-helices and strands. (**B**) shows the multiple sequence alignment of C-terminal region of Rv0367c with ε and its homologues, and the C-terminal of PezA. Structural alignment of both ε and PezA were used to guide the alignment. Putative toxin-binding residue is marked in green and the residue predicted to occlude the nucleotide binding site is shown in blue. Other fully conserved residues are shown in black background, while semi-conserved residues in black boxes. Alignment was generated using ESpript 3.0^60^.

**Figure 5:**
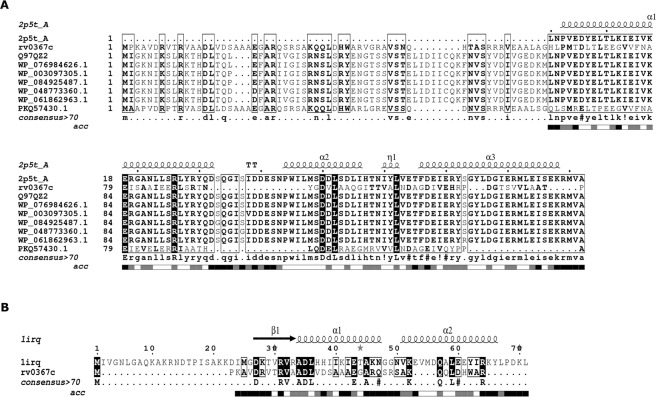
Alignment of PezA^Mtb^ with PezA homologues (**A**) and ω from *S. pyogenes* (**B**). (**A**) Shows alignment of Rv0367c (O53702) with full-length PezA and its homologues. Though the N-terminal region shows sequence conservation, many residues that are a part of the helix-turn-helix motif in the N-terminal of PezA are not conserved in PezA^Mtb^. (**B**) Shows alignment of PezA^Mtb^ (N-terminal) with ω repressor (1rq) of ε/ ζ TA system. Crystal structure of ω was used to guide the alignment. The fully conserved residues between these sequences are highlighted in black. Alignment was generated using ESpript 3.0^60^.

Since we were unable to establish a clear association of the putative antitoxin, Rv0367c, we predicted its fold using Phyre2 and HHpred^40,41^. Here, both methods assigned the RHH domain to the N-terminal region of Rv0367c (Figure S4). PSIPRED, a secondary structure prediction algorithm, also predicted a small ribbon and two helices for the N-terminal region of Rv0367c (Fig. 4A)^42^. RHH domain is a structural motif known to bind to DNA and functions in auto-regulation of many TA modules^43^. In canonical PezA, this regulatory DNA-binding unit is predicted to adopt the HTH motif and is fused to the antitoxin sequence while in ε/ζ/ω TA system, the regulatory protein ω is a separate protein adopting the RHH motif. We additionally performed multiple sequence alignment of the C-terminal region of Rv0367c (residues 40–129) with the C-terminal region of PezA and ε from *S. pneumoniae* and *S. pyogenes*, respectively (Fig. 4B). We observed that the equivalent residues for Leu77/Phe9, Glu84/Glu16, Tyr94/Leu26, Asp98/Leu30, and Asp116/Asn44, which in PezA/ε are important for complex formation, correspond to Val40, Gln47, Val55, Leu59 and Asn77, respectively in Rv0367c. The other solvent exposed and conserved residues, which can function as protein interaction surface in Rv0367c, include Ala50, Ser51, Arg53, Leu63, Asp67, Asp95 and Arg117. In order to determine the extent of sequence similarity in the DNA-binding regions, Rv0367c, full length PezA antitoxin and its homologues were aligned (Fig. 5A). This alignment showed better sequence conservation in the toxin-binding C-terminal than the N-terminal residues. The N-terminal of Rv0367c (residues 1–39), that is predicted to adopt the RHH motif exhibited 30% sequence identity with ω protein from *S. pyogenes* (1irq) in comparison to 10% sequence identity with N-terminal of PezA antitoxin^25,27^. Since the ζ toxins are plasmid-borne and the PezT toxins are their established chromosomal homologues, we predict that Rv0366c-Rv0367c is an unusual PezAT^Mtb^ TA system with N-terminal of PezA^Mtb^ closer to ω.

### Modelled complex structure predicts occlusion of PezT^Mtb^ nucleotide-binding site by PezA^Mtb^ residues

Taking cues from the sequence alignments, we attempted to predict the binding mode of the putative PezAT-like TA system (PezAT^Mtb^). A full-length model was generated for PezA^Mtb^ using Phyre2 intensive mode, using multiple templates (1u9p^44^, 3qoq^45^, 1mnt^40,46^). Though PezA^Mtb^ could be modelled only with 70% confidence, certain secondary structures that can potentially form the interface between the toxin and antitoxin could be predicted with very high confidence. A model of the docked PezT^Mtb^ and PezA^Mtb^ (C-terminal region) was obtained using Z-dock and HADDOCK^47,48^. Since the templates for the TA complex were available (2p5t, 1gvn), restraints were derived based on known interfacial residues in the template that are conserved in the alignments (Fig. 2). Both the programs predicted at least one binding mode with hydrogen bond between Arg116 in PezT^Mtb^ and Gln47 in PezA^Mtb^. This is equivalent to the interaction between Arg157 and Glu84 of PezT and PezA from *S. pneumoniae* and this interaction is important to inhibit toxic activity associated with PezT^25^. The HADDOCK-derived modelled complex structure predicts a binding mode in which, PezA^Mtb^ occludes the nucleotide-binding site along with Asp36 of PezT^Mtb^ (Figure S5).

### Overexpression of PezT^Mtb^ induces bacteriostasis

**Figure 6:**
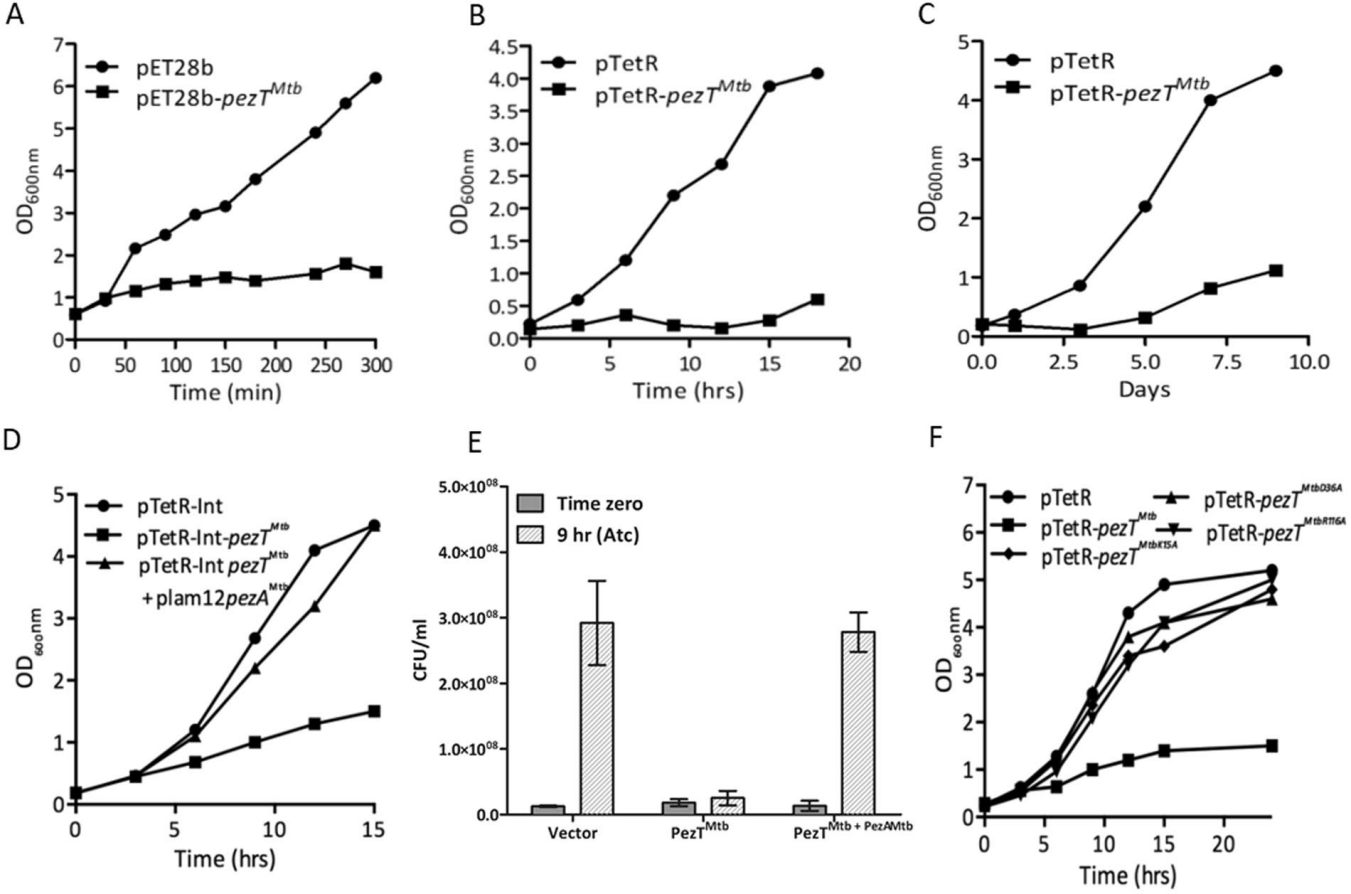
Effect of overexpression of PezT^Mtb^ on bacterial growth. (**A**) *E. coli* BL-21, plysS, λDE3 was transformed with either pET28b or pET28b-*pezT*^*Mtb*^. The expression of PezT^Mtb^ was induced by addition of 1 mM IPTG and growth was monitored by measuring OD_600nm_ at indicated time points. (**B**,**C**) Expression of PezT^Mtb^ in both *M. smegmatis* mc^2^155 and *M. tuberculosis* H_37_Rv was induced by addition of 50 ng/ml anhydrotetracycline in liquid cultures. The growth of induced cultures was monitored by measuring OD_600nm_. (**D**,**E**) For co-expression studies, the expression of PezT^Mtb^ and PezA^Mtb^ was induced by addition of 50 ng/ml anhydrotetracycline and 0.2% acetamide, respectively. The growth of induced cultures was monitored by measuring OD_600nm_ (**D**) and viable counts (**E**). For bacterial enumeration 10.0-fold serial dilutions were prepared and plated on MB7H11 plates. The plates were incubated at 37 °C for 2–3 days. The data shown in this panel is representative of two independent experiments. (**F**) *M. smegmatis* strains harbouring vector or PezT^Mtb^ or PezT^MtbK15A^ or PezT^MtbD36A^ or PezT^MtbR116A^ were grown in MB7H9 medium till OD_600nm_ of 0.2. The effect of expression of wild type and mutant PezT^Mtb^ proteins on *M. smegmatis* growth was monitored by measuring OD_600nm_. The data shown in these panels is representative of three independent experiments.

In order to functionally characterize PezT^Mtb^, growth inhibition upon overexpression of PezT^Mtb^ in both *E. coli* and mycobacterial strains was studied. The expression of Rv0366c in BL-21 (λDE3, plysS) was induced by the addition of 1 mM isopropyl β-D-1-thiogalactopyranoside (IPTG). We observed that overexpression of PezT^Mtb^ resulted in severe growth inhibition of *E. coli* (Fig. 6A). As expected, no growth inhibition was seen in *E. coli* harbouring vector control (Fig. 6A). To investigate the effect of PezT^Mtb^ overexpression in fast growing (*M. smegmatis*) and slow growing (*M. tuberculosis*) bacteria, it was cloned in an anhydrotetracycline-based inducible vector, pTetR^49^. Consistent with *E. coli* results, inducible expression of PezT^Mtb^ inhibited growth of both *M. smegmatis* and *M. tuberculosis* H_37_Rv (Fig. 6B,C). We also noticed that this growth inhibition was restored by co-expressing PezT^Mtb^ along with the predicted antitoxin partner, PezA^Mtb^ (Fig. 6D). As shown in Fig. 6E, we observed that PezT^Mtb^ overexpression resulted in bacteriostatic effect as reported earlier in the case of other type II toxins such as RelE, MazF and VapC^23,49–52^. To further characterize PezT^Mtb^, we performed growth inhibition experiments with *M. smegmatis* overexpressing Rv0366c harbouring mutations in the predicted catalytically important residues such as Asp36, Lys15 and Arg116 (Fig. 6F). As expected, mutations of these residues to either alanine or valine abrogated the growth inhibition activity associated with PezT^Mtb^.

### Overexpression of PezT^Mtb^ reduces cell length and increases tolerance of *M. smegmatis* to ethambutol *in vitro*

**Figure 7:**
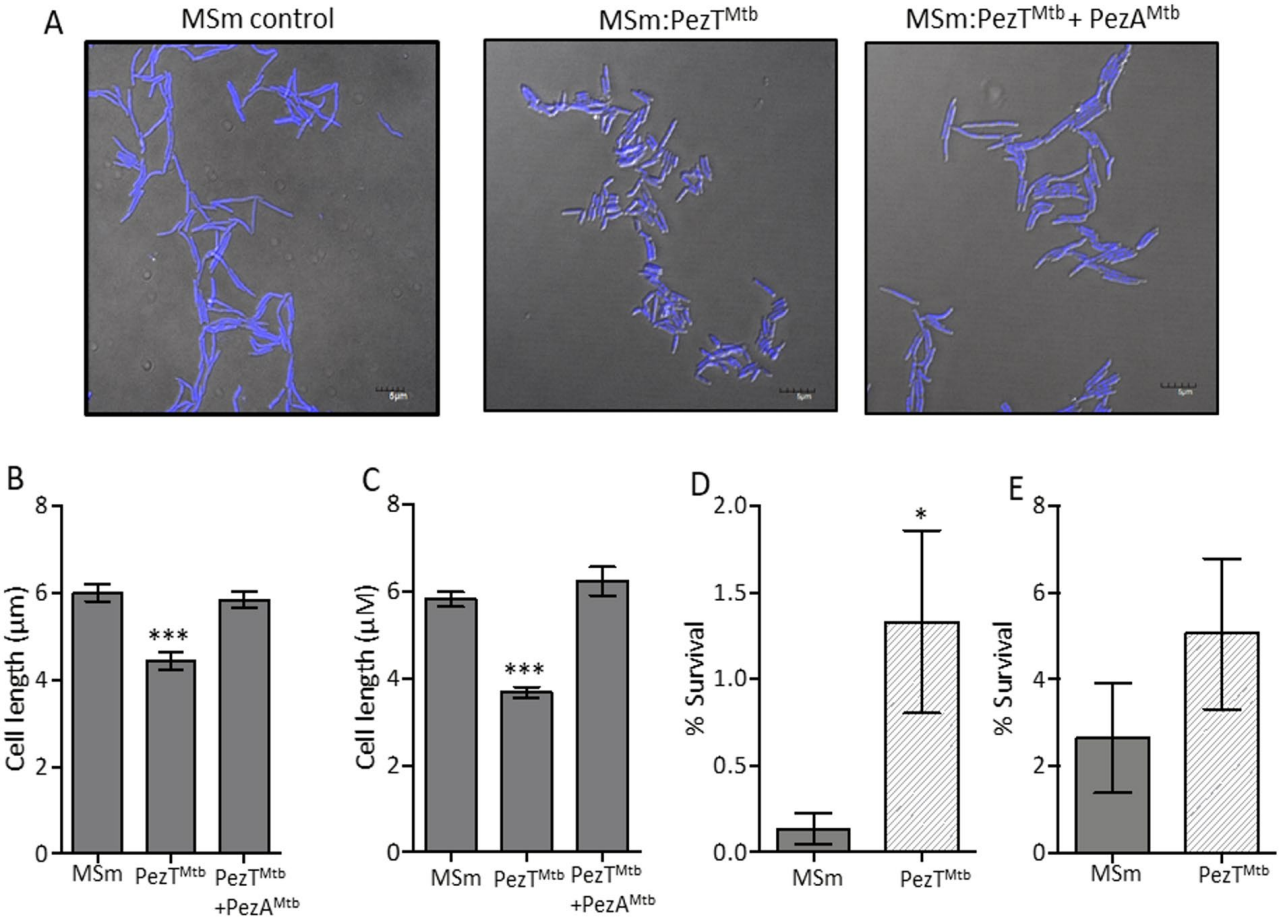
Effect of PezT^Mtb^ overexpression on cell length and drug tolerance of *M. smegmatis*. (**A**) DAPI stained fixed images of *M. smegmatis* harbouring vector or expressing PezT^Mtb^ in the absence or presence of PezA^Mtb^ after 3 hrs of induction. (**B**,**C**) The panel depicts cell length of *M. smegmatis* harbouring either pTetR-Int or pTetR-Int-PezT^Mtb^ or pTetR-Int-PezT_Mtb_ and pLam12-PezA^Mtb^. The cultures were induced for either 3 hrs (**B**) or 6 hrs (**C**) and bacilli length was measured using Fluoview FV1000 software. The y-axis represents mean ± S.E. of cell length for bacilli per strain. The data shown in this panel is representative of three independent experiments. (**D**,**E**) Induced *M. smegmatis* cultures harbouring either pTetR-Int or pTetR-Int-PezT^Mtb^ were exposed to either 15.6 μM ethambutol or 2.0 μM levofloxacin. For bacterial enumeration, 12 hrs post-exposure, cultures were harvested, washed and 100 μl of 10.0-fold serial dilutions were plated on MB7H11 plates at 37 °C for 2–3 days. Significant differences were observed for the indicated groups (paired, two-tailed, t-test, *P < 0.05, ***P < 0.001).

There are accumulating evidences that morphological changes occur in bacteria upon exposure to different stress conditions. We have shown earlier that overexpression of VapC toxins resulted in bacterial population that possessed a bulge^49^. Previously, it has been shown that overexpression of PezT results in cell wall lysis and formation of mid-cell positioned bulges in *E. coli*^53^. Hence, we next compared the cell morphology and length of vector harbouring and PezT^Mtb^ overexpressing *M. smegmatis* strains. As shown in Fig. 7A, we noticed that 4′,6-diamidino-2-phenylindole (DAPI) uniformly stained the nucleoid in both strains. Interestingly we observed that length of bacilli in PezT^Mtb^ overexpression strain was reduced in comparison to the parental strain (Fig. 7A). The bacilli in both parental and overexpression strains at 3 hrs and 6 hrs post-induction appeared as rod shaped (Fig. 7A). We observed that the average length of bacteria in PezT^Mtb^ overexpression strain was 4 μm in comparison to 6 μm in strain harbouring vector control (Fig. 7B,C). As expected these differences were not observed in *M. smegmatis* upon overexpression of PezT^Mtb^ toxin along with its cognate PezA^Mtb^ antitoxin (Fig. 7A–C). The observed reduction in bacilli length upon overexpression of PezT^Mtb^ in *M. smegmatis* was observed to be statistically significant (***p < 0.001). We also observed that the cell length of *M. smegmatis* overexpressing mutant proteins was similar to that observed for the parental strain (Figure S6). Further, we determined the contribution of PezT^Mtb^ to the formation of drug-tolerant persisters. We quantified the fraction of persisters in early-log phase *M. smegmatis* overexpression or vector control cultures upon exposure to either ethambutol or levofloxacin. As shown in Fig. 7D, ethambutol exposure increased the number of drug-tolerant persisters by 10.0-fold compared to empty vector control (*p < 0.05). However, this increase in tolerance was not observed in cultures upon exposure to levofloxacin (Fig. 7E). The number of levo-tolerant persisters were comparable in *M. smegmatis* PezT^Mtb^ overexpressing or vector harbouring strains. The percentage of surviving bacteria varied between 2.0–4.0% after 24 hrs post-drug exposure (Fig. 7E). These results are in concordance with previous studies demonstrating that overexpression of RelE contributes to increase in drug-specific persisters *in vitro*^50^.

### Conservation of PezAT-like system of H_37_Rv in other mycobacterial species

**Figure 8:**
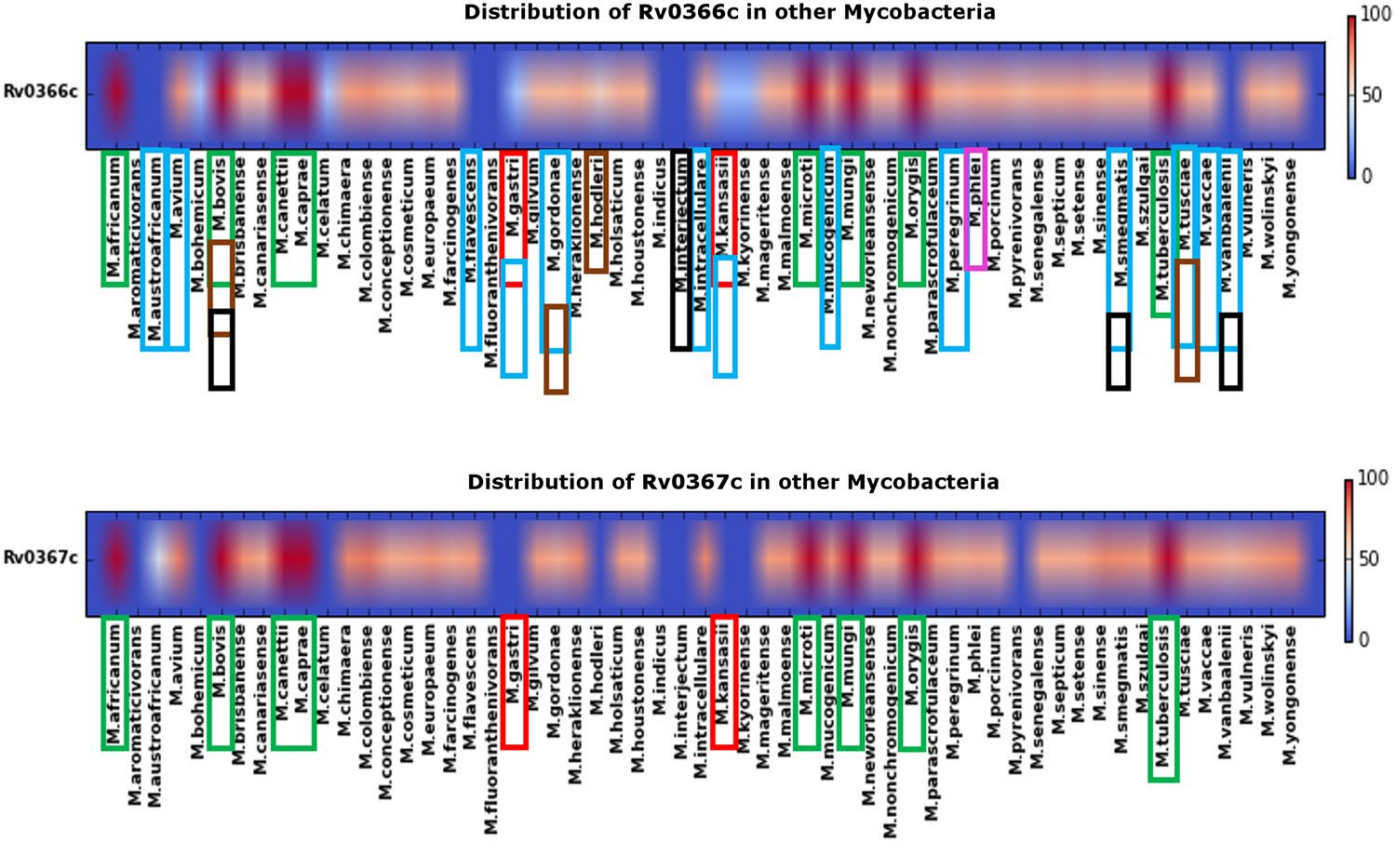
Distribution of PezT^Mtb^ (upper panel) and PezA^Mtb^ (lower panel) in the genus Mycobacterium. The hits were obtained at an e-value better than 1e^−04^ and query coverage better than 70% from TBLASTN. The organisms belonging to MTBC are marked in green in both panels. Red marking indicates the organisms in which maximum number of other H_37_Rv toxin-antitoxin systems have been found to be conserved (unpublished). The other colours represent the habitat that these organisms belong to: cyan – water, brown – soil, pink – plants, black – air.

The presence of a non-canonical PezAT^Mtb^ prompted us to probe for the prevalence of such a non-canonical system in 101 other mycobacterial genomes using TBLASTN (Fig. 8). We observed that homologs of either PezT^Mtb^ or PezA^Mtb^ were present in 57 mycobacterial species while no hits were identified in the remaining 44 mycobacterial genomes. Out of the 57, these proteins existed as a TA pair in 20 bacterial genomes. Interestingly, a majority of these TA pairs are present in pathogenic bacteria such as the MTBC (marked in green) and shared a sequence identity of greater than 80% amongst themselves (Fig. 8). Others such as *Mycobacterium avium, Mycobacterium chimaera, Mycobacterium gordonae, Mycobacterium mucogenicum*, and *Mycobacterium peregrinum* also possess homolog for PezAT^Mtb^ and are non-tuberculosis bacteria capable of causing various human diseases. We also noticed that homolog of PezT^Mtb^ is present in *Mycobacterium gastri* and *Mycobacterium kansasii* with a sequence identity lower than 40%. Similarly, in other non-pathogenic bacteria like *Mycobacterium austroafricanum*, *Mycobacterium vanbaalenii* etc. the homologs of either the PezT^Mtb^ toxin or PezA^Mtb^ antitoxin exist but with poor sequence identity (Fig. 8). A separate search of the canonical UNAG-binding ζ toxins in mycobacterial species does not provide any noteworthy results, further strengthening the observation that the genus Mycobacterium may harbour only such non-canonical systems.

### Probing the presence of PezAT^Mtb^ system outside genus *Mycobacterium*

The classical ε/ζ/ω or PezAT systems are ubiquitously present in Gram-positive as well as Gram-negative bacteria^36^. Hence, we investigated, whether this non-canonical PezAT^Mtb^ system also occurs in other prokaryotes. To address this question, we searched for its homologues in nearly 4500 bacterial and archaebacterial representative genomes using TBLASTN (Table 1). Interestingly, only 26 organisms outside *Mycobacterium* possess homologues for the PezAT^Mtb^ pair. Majority of these organisms belong to the family Gordoniaceae and Nocardiaceae which are members of the order Corynebacteriales as is Mycobacteriaceae. *Kineosphaera limosa*, which belongs to the same class as *Mycobacterium*, also possesses a homolog of this non-canonical system. In all these organisms, the putative toxin is annotated as ATPase while the upstream putative antitoxin is a hypothetical protein. A separate search for canonical ε/ζ/ω or PezAT systems (1gvn and 2p5t) found homologs mainly in families Enterococcaceae and Streptococcaceae, belonging to class Bacilli, but no hits were observed in the organisms where the homologs of PezAT^Mtb^ system were found. This analysis shows that non-canonical PezAT^Mtb^ system are largely confined to the order Corynebacteriales with no homologues in organisms outside this order.

**Table 1:** Distribution of homologues of Rv0366c and Rv0367c, as pair, in organisms outside genus *Mycobacterium*.

S. no	Name of the organism	Order
1	*Amorphus coralli*	Rhizobiales
2	*Brevibacterium linens*	Corynebacteriales
3	*Conexibacter woesei*	Solirubrobacterales
4	*Corynebacterium-like bacterium*	Corynebacteriales
5	*Gordonia aichiensis*	Corynebacteriales
6	*Gordonia araii*	Corynebacteriales
7	*Gordonia desulfuricans*	Corynebacteriales
8	*Gordonia hirsute*	Corynebacteriales
9	*Gordonia jacobaea*	Corynebacteriales
10	*Gordonia malaquae*	Corynebacteriales
11	*Gordonia polyisoprenivorans*	Corynebacteriales
12	*Gordonia rhizosphere*	Corynebacteriales
13	*Gordonia terrae*	Corynebacteriales
14	*Hydrocarboniphaga effuse*	Nevskiales
15	*Kineosphaera limosa*	Micrococcales
16	*Novosphingobium barchaimii*	Shingomonodales
17	*Oceanicaulis alexandrii*	Rhodobacteriales
18	*Parvibaculum lavamentivorans*	Rhizobiales
19	*Rhodococcus fascians*	Corynebacteriales
20	*Rhodococcus jostii*	Corynebacteriales
21	*Rhodococcus pyridinivorans*	Corynebacteriales
22	*Rhodococcus triatomae*	Corynebacteriales
23	*Roseivivax atlanticus*	Rhodobacteriales
24	*Salinisphaera shabanensis*	Salinisphaerales
25	*Smaragdicoccus niigatensis*	Corynebacteriales
26	*Tomitella biformata*	Corynebacteriales

## Discussion

The reasons for the expanded repertoire of TA systems in organisms like *M. tuberculosis* remain a mystery. While it is speculated that many have been acquired through lateral gene transfer and gene duplication, the functional implications of such an expanded repertoire have been suggested as beneficial to bacterial pathogenesis, virulence and survival mechanisms^54^. Although various type II TA systems have been identified in *M. tuberculosis*, this study reports for the first time, the identification and characterization of a PezAT-like TA system in this organism. PezT and its plasmid homologues ζ toxins that have been demonstrated to phosphorylate UNAG function as nucleotide kinases. The phosphorylated form of UNAG inhibits MurA and other enzymes involved in peptidoglycan biosynthesis and maintenance of cell wall integrity^25,28,30^. The homologue in *M. tuberculosis* was identified through computational searches involving many canonical and non-canonical ζ and PezT toxins. Since the homologue was not plasmid encoded, we annotated Rv0366c as PezT^Mtb^. This *M. tuberculosis* homologue harboured antitoxin-binding arginine residue (at position 116), an aspartate residue (at position 36) that prepares the substrate for phosphorylation through deprotonation, and the P-loop motif for NTP binding. However, the residues of ζ and PezT toxins that bind to known substrates such as UNAG were not conserved in PezT^Mtb^.

Our searches for homologues of the canonical antitoxin using ε/PezA were less successful in identifying the cognate antitoxin due to two reasons: a) extensive sequence dispersion of the antitoxin and b) differences in the DNA-binding regulatory region of the antitoxin. Most antitoxins are composed of an N-terminal DNA binding region and a C-terminal toxin-binding region: two functions encoded in a single protein. However, in ε/ζ/ω system, ε possess only the toxin-binding region and ω encodes a separate protein that participates in DNA-binding and regulation. In the PezAT^Mtb^ TA system that we have identified in *M. tuberculosis*, the N-terminal of PezA^Mtb^ shares 30% sequence identity with ω protein that binds DNA in the ε/ζ/ω TA pair. Its C-terminal shares 17% sequence identity to the canonical, chromosomally encoded, PezA- antitoxins. Therefore, the non-canonical PezAT^Mtb^ TA that we report here shows signatures that are characteristic of both the ε/ζ/ω and PezAT TA families.

We further show that overexpression of PezT^Mtb^ toxin induces bacteriostasis in various bacterial systems and co-expression of PezA^Mtb^ antitoxin could rescue this growth defect. The inhibition of toxic activity of PezT^Mtb^ by co-expression of the PezA^Mtb^ antitoxin in *M. smegmatis* and *E. coli* corroborates that the Rv0366c-Rv0367c locus encodes a TA pair. Indeed, their binding modes, predicted through docking studies, proposes a complex that is similar to known PezAT and ε/ζ/ω TA systems. As reported in the abundant ζ toxins of Gram-positive bacteria, here also the antitoxin binds in a manner that occludes the nucleotide-binding pocket of the toxin. Contrary to published reports in *E. coli*, we did not observe any bulge formation in PezT^Mtb^ overexpression *M. smegmatis* strains^53^. The findings that the presence of free PezT^Mtb^ results in decreased bacterial length is suggestive of the fact that phosphorylation of UNAG might not be the mechanism by which PezT^Mtb^ inhibits bacterial growth. A homologue from *X. oryzae* (AvrRxo1), is known to phosphorylate coenzyme NAD and its precursors^39^. More recently, a hitherto unknown homologue from *N. gonorrhoeae* that targets UDP-activated sugars, resulting in cell lysis, has also been characterized^30^. However, an alignment of PezT^Mtb^ with the *N. gonorrhoeae* ζ toxin and *X. oryzae* (AvrRxo1) shows conservation of only few of the substrate-binding residues, mainly with AvrRxo1. We reckon therefore, that PezT^Mtb^, which does not show high conservation of UNAG-binding residues, might target NAD or an altogether different cellular substrate. However, more detailed experiments will be required to further characterize the enzymatic activity associated with PezT^Mtb^. We also show that ectopic expression of PezT^Mtb^ results in a metabolic state that results in drug-tolerance specific to ethambutol. These observations are in concordance with previous studies where the effect of overexpression of toxins on mycobacterial tolerance has been shown to be drug-specific^50^.

Recently, Jaén-Luchoro and co-workers reported the presence of a putative ζ toxin in the sequenced genome of an isolate of Mycobacterium MSDH3, a close homologue of *Mycobacterium chelonae*^55^. While the toxin sequence in their study bears considerable similarity with the known ζ toxins, no conclusive functional annotation was made for the antitoxin sequence. They reported remote similarities with the ParD antitoxin and shared similarities with the Arc repressor Pfam domain family whose members possess the RHH DNA-binding motif. We aligned the proteins reported in their study to PezAT^Mtb^ (Figure S7) and found that PezT^Mtb^ and PezA^Mtb^ share high sequence identity with the MHSD3 systems. Our study has attempted a more detailed annotation of the antitoxin sequence and we find that it shows high similarity with the ω of the ε/ζ/ω toxin antitoxin system. While they limited their analysis to assessments of similarities with close hits detected in BLAST searches, we have performed a more exhaustive survey for the presence of such a TA system in all mycobacteria and extended the searches to nearly 4500 prokaryotic genomes. Indeed, our comparative study shows that homologues of the PezAT^Mtb^ were restricted to the order Corynebacteriales.

A separate search for homologues of classical ε/ζ/ω systems finds hits in the genus *Enterococcus, Streptococcus* and *Lactococcus* but no significant hits in *Mycobacterium*. Nearly 20 pathogenic mycobacterial species show conservation of the non-canonical PezT^Mtb^ as pairs. Although further experimental evidence will be required to demonstrate the cellular targets of this non-canonical TA pair and its role in conferring virulence to H_37_Rv, its homology to proteins from pathogenic bacteria raises the possibility that PezAT^Mtb^ might also be implicated in bacterial pathogenesis and virulence. A search for homologues of this system in species outside the genus *Mycobacterium* revealed that it is conserved in a few micro-organisms outside Mycobacteriaceae family. Interestingly these families belong to the order Actinomycetales that includes the genus *Mycobacterium*. This strengthens the idea that this non-canonical PezAT-like system is largely confined to the class Actinobacteria and in fact mainly to Mycobacteriaceae. The results presented in the study indicate that this system has likely undergone selective evolutionary pressure to remain confined within this class of bacteria. Taken together, this study reports the identification, characterisation and conservation analysis of a non-canonical PezAT-like TA system in *M. tuberculosis*. Future experiments would aim i) to understand the plausible mechanism of growth inhibition by PezT^Mtb^ and (ii) construction of knock out strains to determine the essentiality of this TA pair *in vivo*.

## Experimental Procedures

### Search for ζ/PezT toxin homologue in *M. tuberculosis* H_37_Rv

Putative ζ toxin was searched in *M. tuberculosis* using two parallel approaches. In the first approach, 1036 ζ toxin sequences from the Pfam database were queried in the *M. tuberculosis* genome using TBLASTN^32,33^. These queries included both experimentally validated as well as predicted ζ and PezT toxin sequences from various organisms. In addition, atypical ζ toxin-like sequences that do not have a conserved UNAG-binding motif (GTXR) were also employed as queries. To increase the search sensitivity, other bacterial genome sequences were also included in the search database. Hits were filtered using an e-value cut-off of 1e^−06^ and a query coverage of 60%. Separately, Pfam domains were also assigned to the *M. tuberculosis* proteome, using the PfamScan program in Pfam, to identify proteins with the ζ toxin domain.

### Identification of ε/PezA antitoxin homologue in *M. tuberculosis* H_37_Rv

After the identification of putative ζ toxin loci in *M. tuberculosis*, the neighbouring genes of each hit were analysed to search for a cognate antitoxin. The neighbouring gene was considered as the putative antitoxin only if (1) it precedes the putative toxin and (2) its sequence distance from the putative toxin gene is ≤150 bp^23^. Pfam domains were assigned to each such putative antitoxin to scan for the ε/PezA antitoxin domain. As in the search for the toxins, an independent sequence-based search for ε/PezA sequences from the Pfam database was also performed in the *M. tuberculosis* proteome, using TBLASTN. Rv0367c was identified as the putative antitoxin sequence and aligned with ε and PezA antitoxin sequences from the Pfam database, using MAFFT, to predict toxin-binding residues^35^. As for the toxin, a homology model was derived for the putative antitoxin, using templates identified by Phyre2^40^.

### Prediction of putative toxin structure and antitoxin-binding residues

Rv0366c was predicted as a homologue of the ζ and PezT toxins from our query dataset. It was modelled with modeller using the templates identified in Phyre2 and HHpred searches^40,41^. 1gvn is a ζ toxin from *S. pyogenes* and 2p5t is a PezT toxin from *S. pneumoniae*. Both templates show a Phyre2 confidence score >99% and share 18% and 20% sequence identity with Rv0366c. A structure-guided alignment, derived using Promals3D, was employed to build the model^56^. 100 models were generated and the model with the least discrete optimized protein energy (DOPE) score was selected. MAFFT was employed to align Rv0367c with its homologous sequences from the Pfam database. Conserved, surface-exposed residues were recognized from the multiple sequence alignment and mapped onto the modelled structure, to evaluate the compatibility of its interaction interfaces with the antitoxin or substrate.

### Bacterial strains, plasmids and growth conditions

All bacterial strains and plasmids used in the study are listed in Table S2. Bacterial strains were grown with shaking at 200 rpm, 37 °C in Luria Bertani medium (LB) for *E. coli* or Middlebrook MB7H9 medium for mycobacteria. The growth of cultures was monitored by either spotting or measuring optical densities at 600 nm. When required, antibiotics were used at the following concentrations; kanamycin (25 μg/ml for *E. coli*), ampicillin (100 μg/ml for *E. coli*), hygromycin (150 μg/ml for *E. coli*, 50 μg/ml for mycobacteria) and tetracycline (10 μg/ml for *E. coli*). The sequences of oligonucleotides used in the study are shown in Table S3.

### DNA manipulations

DNA manipulations and other molecular biology techniques were performed according to standard protocols. The ORF encoding Rv0366c was amplified from *M. tuberculosis* genomic DNA using primers Rv0366c-F and Rv0366c-R. The PCR amplicons were digested and cloned into either IPTG inducible pET28b (for *E. coli* studies) or pTetR or pTetR-Int (for mycobacterial studies^49^). The recombinant pET28b-PezT^Mtb^ was confirmed by sequencing and transformed into BL-21 (λDE3, plysS) and expression was induced by addition of 1 mM IPTG. Recombinant pTetR constructs were electroporated into either *M. smegmatis* mc^2^155 or *M. tuberculosis* H_37_Rv and transformants were selected on MB7H11 medium plates supplemented with hygromycin. The ORF encoding for antitoxin, Rv0367c was PCR amplified using primers Rv0367c-F and Rv0367c-R and cloned into acetamide inducible vector plam12^57^. Further, the hygromycin resistance gene was replaced with kanamycin resistance gene resulting into plam-Rv0367c-kan. The construct was sequenced prior to transformation in mc^2^155 harbouring an integrative copy of PezT^Mtb^. Positive transformants were selected on MB7H11 plates supplemented with kanamycin and hygromycin.

### Activity of PezT^Mtb^ in *E. coli* and mycobacteria

For growth inhibition assays in *E. coli*, expression of PezT^Mtb^ was induced by the addition of 1 mM IPTG. For overexpression of PezT^Mtb^ in *M. smegmatis* and *M. tuberculosis*, cultures were grown till OD_600nm_ of 0.2 and toxin expression was induced by the addition of 50 ng/ml anhydrotetracycline. For co-expression studies, the expression of antitoxin was induced by the addition of 0.2% acetamide. The experiments involving *M. smegmatis* recombinant strains were performed in standard Bio-safety level 2 containment facilities. The growth curves using various *M. tuberculosis* strains were carried out in Bio-safety level 3 containment facilities.

### Drug tolerance assays in *M. smegmatis*

For these experiments, expression of toxins in *M. smegmatis* was induced by the addition of 50 ng/ml anhydrotetracycline. *M. smegmatis* strains harbouring pTetR or pTetR-PezT^Mtb^ were diluted to OD_600nm_ of 0.05 in medium containing either ethambutol (cell wall inhibitor) or levofloxacin (DNA replication inhibitor) and incubated at 37 °C for 24 hrs. For bacterial enumeration, 10.0-fold serial dilutions were prepared and 100 μl was plated on MB7H11 plates in duplicates. Percentage survival was calculated from the mean colony-forming unit (CFU) obtained in the culture after drug incubation divided by CFU before the addition of drug.

### Cell length experiments

In order to investigate the effect of PezT^Mtb^ overexpression on cell length, toxin expression in *M. smegmatis* was induced by the addition of 50 ng/ml anhydrotetracycline for either 3 hrs or 6 hrs. At designated time points, induced cultures were harvested, washed, fixed with 4% paraformaldehyde and stained with DAPI as previously described^49^. DAPI stained bacilli were viewed using FV1000 confocal microscope (Olympus, Japan). The morphology and length of bacteria was visualised and analysed using fluoview software (Olympus, Japan).

### Distribution of putative *M. tuberculosis* PezAT-like TA system in Mycobacteria and other prokaryotes

TBLASTN searches were performed, by querying for homologues of PezT^Mtb^ and PezA^Mtb^ against a database consisting of 101 mycobacterial and 4500 other bacterial and archaebacterial genomes. A cut-off of 1e^−04^ and a query coverage of 70% was applied to filter the hits. Care was taken to consider only those homologues of PezT^Mtb^ and PezA^Mtb^ that were not separated by >150 bp in various genomes.

## Supplementary information

Supplementary information

## Data Availability

All data pertaining to the manuscript have been provided in the form of figures. Supporting information has been made available as Tables S1-S3 and supporting Figures S1–S7. Datasets pertaining to the sequence searches described here are available from the corresponding author on reasonable request.
