# Supplementary material for: Mycobacterium tuberculosis Rv0366c-Rv0367c encodes a non-canonical PezAT-like toxin-antitoxin pair
Source: Sci Rep. 2019 Feb 4;9:1163. doi: 10.1038/s41598-018-37473-y (PMC6362051; doi:10.1038/s41598-018-37473-y)
Supplement: Supplementary file 1 — Supplementary information [file 41598_2018_37473_MOESM1_ESM.pdf]

Mycobacterium tuberculosis Rv0366c-Rv0367c encodes a non-canonical PezAT-like toxin-antitoxin pair

Himani Tandon[1#], Arun Sharma[2#], Sankaran Sandhya[1], Narayanaswamy Srinivasan[1*], Ramandeep Singh[2*]

From the [1]Molecular Biophysics Unit, Indian Institute of Science, Bangalore-560012; [2] Tuberculosis Research Laboratory, Vaccine and Infectious Disease Research Centre, Translational Health Science and Technology Institute, NCR Biotech Science Cluster, Faridabad, Haryana- 121001, India,

[#]Joint first authors

* To whom correspondence should be addressed: Narayanaswamy Srinivasan, Molecular Biophysics Unit, Indian Institute of Science, Bangalore-560012;
ns@iisc.ac.in; Tel: +918022932837; Fax: +918023600535;

Ramandeep Singh, Vaccine and Infectious Disease Research Centre, Translational Health Science and Technology Institute, NCR Biotech Science Cluster, Faridabad, Haryana-121001, India;
ramandeep@thsti.res.in; Tel: +91-129-2876305, Fax: +91-129-2876402

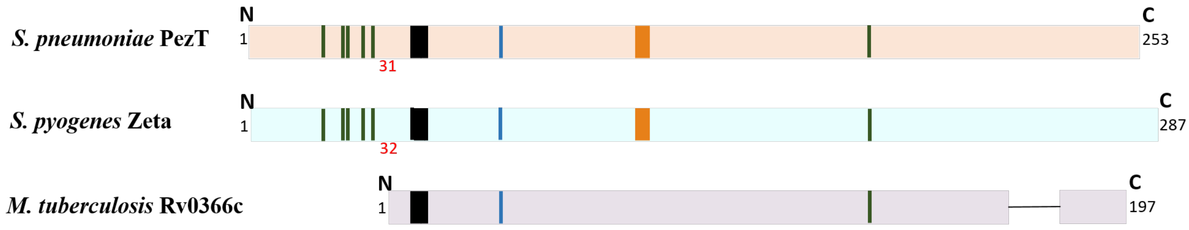

Figure S1. **Schematic representation for PezT, ζ and Rv0366c.** A comparison of Rv0366c length with canonical PezT from *S. pneumoniae* and ζ from *S. pyogenes* toxins is shown. Rv0366c is shorter by ~80 residues when compared to the other two proteins. It does not align to the N-terminus region (1-32 residues), common to PezT and ζ. The N-terminus in both PezT and ζ is known to harbour few antitoxin binding residues, shown in green bars. The P-loop motif (in blue bar) and one antitoxin binding residue (in green bar) is conserved between the three. The UNAG binding site (in orange) is not conserved in Rv0366c.

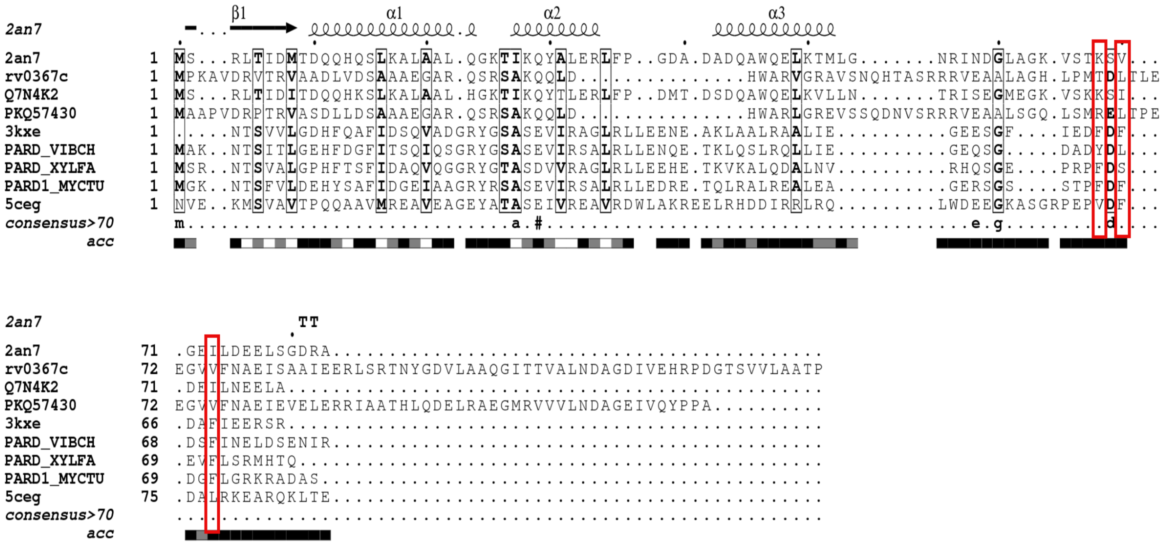

Figure S2. Alignment of Rv0367c with ParD antitoxin family. A conservation of the N-terminus RHH DNA binding motif of PezA^{Mtb} and ParD antitoxins was observed. The remaining residues, including toxin binding residues in ParD (highlighted in red), are not very well conserved in Rv0367c.

A

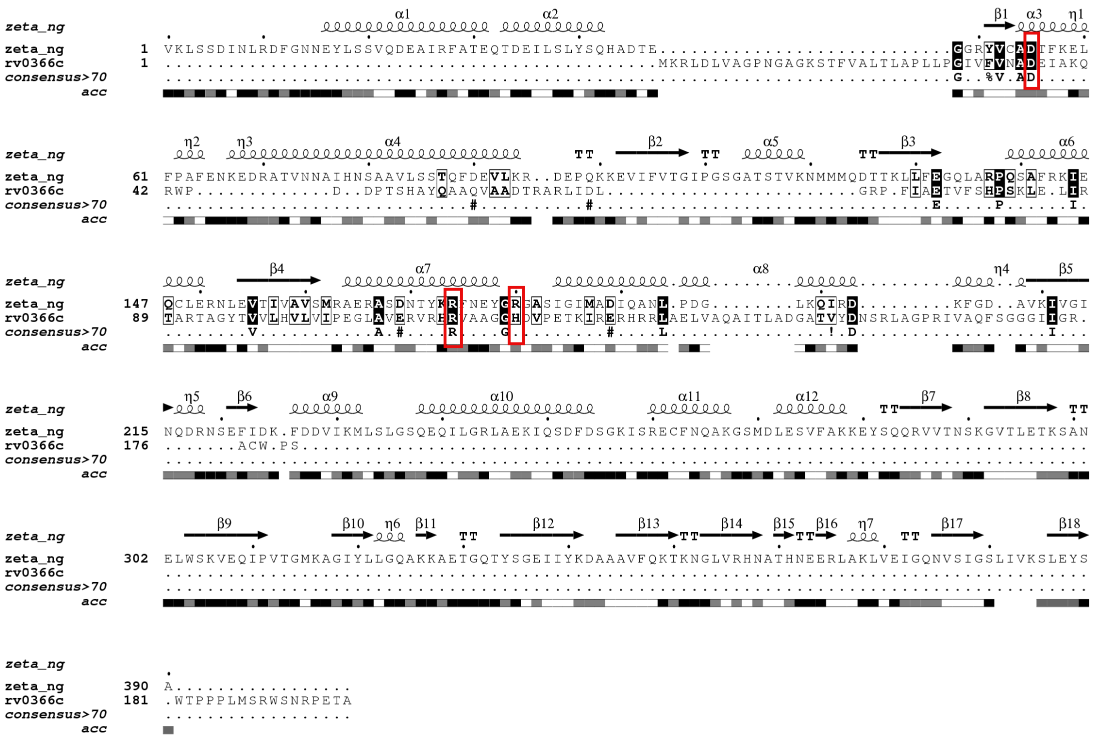

B

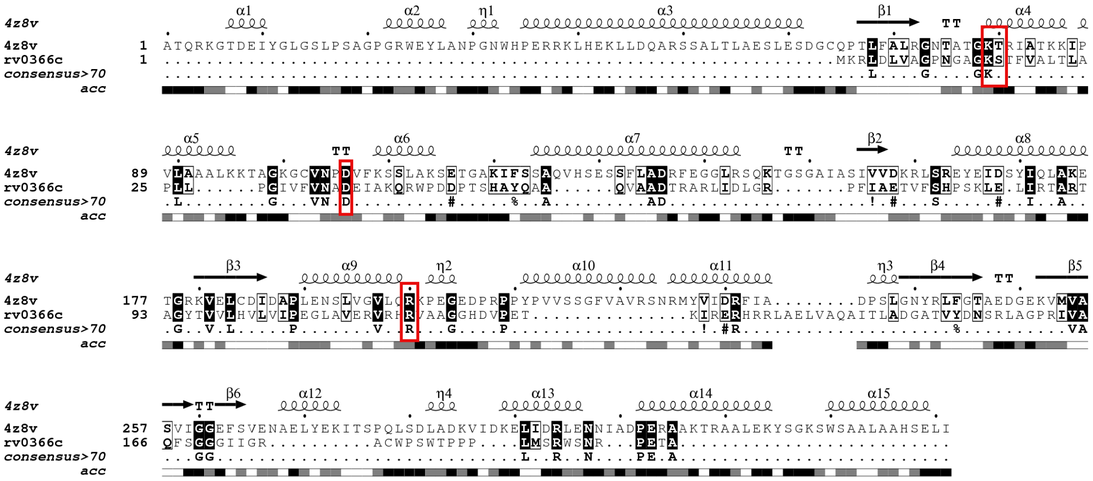

Figure S3. Alignment of Rv0366c with recently reported ζ toxins. (A) Alignment between *Neisseria gonorrhoeae* ζ (ζ_ng) and Rv0366c shows conservation of 2 residues known to bind UNAM or UNAM-4P (in red box). **(B)** Alignment between *Xanthomonas Oryzae pv. oryzicola* AvrRxo1 and Rv0366c shows conservation of 4 catalytic residues (in red box).

```
  >1baz_A ARC repressor; transcription regulation; 1.90A {Enterobacteria phage P22} SCOP: a.43.1.1 PDB:  1bdv _A*
 1arq _A  1arr _A  1bdt _A*  1par _A*  1myk _A  1qtg _A  1b28 _A  1myl _A
Probab=97.01  E-value=0.0019  Score=38.00  Aligned_cols=38  Identities=13%  Similarity=0.126  Sum_probs=0.0

Q ss_pred              CccccccEEeCHHHHHHHHHHHHHccCCHHHHHHHHHH
Q M.              2 PKAVDRVTRVAADLVDSAAAEGARQSRSAKQQLDHWAR   39 (129)
Q Consensus       2 ~~~~~~~~vrl~~~L~~~a~~~a~~~~RS~~~Qie~War   39 (129)
                    ..+...++||+++|++..+..|+...||+++.|..++.
T Consensus       5 ~~~~~~~~irl~~~l~~~l~~~A~~~g~S~s~~i~~~l~   42 (53)
T 1baz_A         5 SKMPQVNLRWPREVLDLVRKVAEENGRSVNSEIYQRVM   42 (53)
T ss_dssp              CCSCEEEEEECCHHHHHHHHHHHHHTTCCHHHHHHHHHH
T ss_pred              CCCceEEEECCHHHHHHHHHHHHHhCCCHHHHHHHHHH
```

Figure S4. Fold assignment to the NH_2-terminal region of Rv0367c using HHpred. The template structures identified by HHpred have an RHH domain.

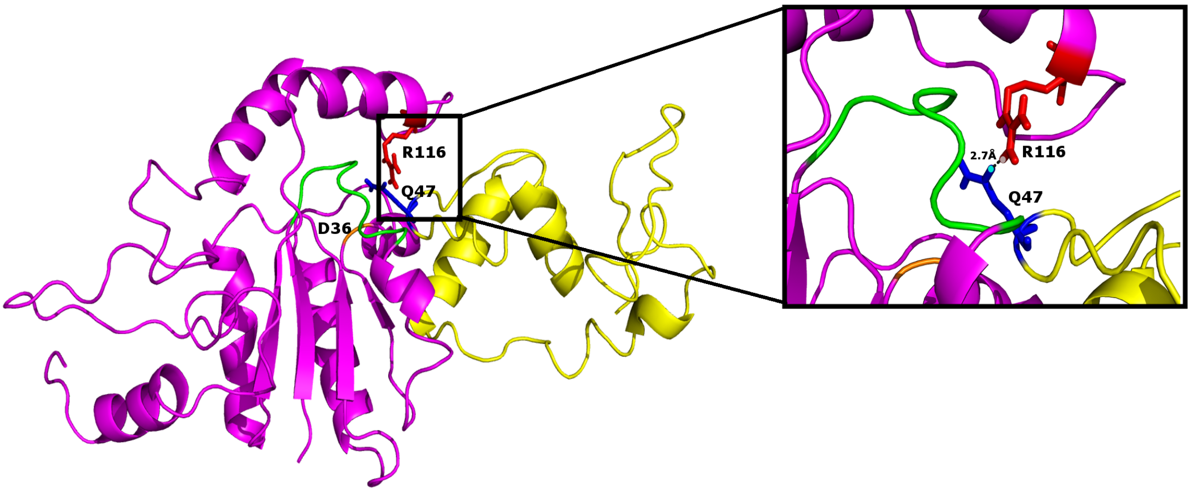

Figure S5. Binding mode for Rv0366c-Rv0367c (PezAT^{Mtb}) as predicted by HADDOCK. The information on the putative binding residues for the complex was gathered from the multiple sequence alignments and was provided to HADDOCK prior to docking. This model shows R116 in Rv0366c, in red and Q47 in Rv0367c, in blue. D36 is shown in orange and nucleotide binding region, in green. The distance between H atom of the NH2 moiety of R116 (brown) and OE1 atom of Q47 (cyan) in this model is 2.7 Å suggesting a potential hydrogen bond. This model also shows the occlusion of nucleotide binding region by PezA^{Mtb}.

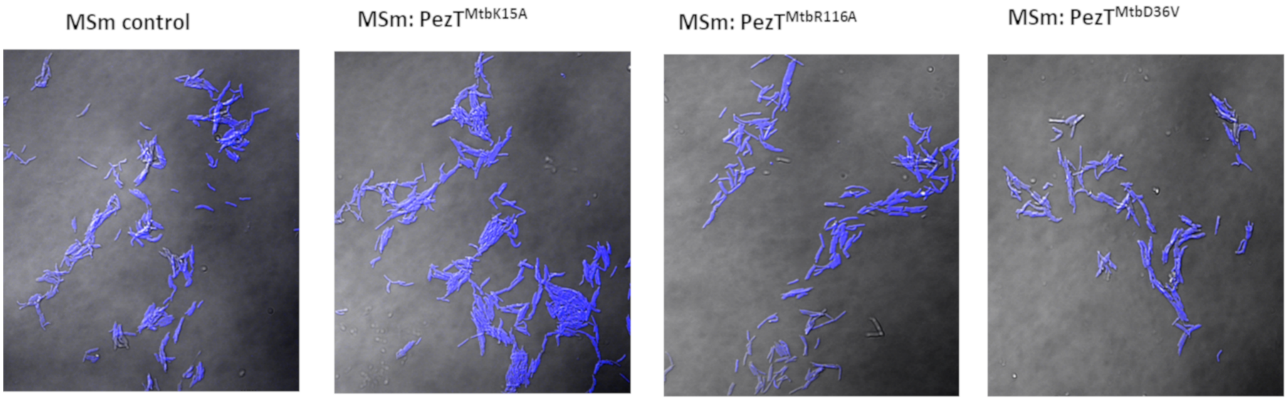

Figure S6. Effect of overexpression of PezT^{Mtb} single mutants on cell length of *M. smegmatis*. DAPI stained fixed images of *M. smegmatis* harbouring vector or expressing PezT^{MtbK15A} or PezT^{MtbR116A} or PezT^{MtbD36V} after 6 hrs of induction. The data shown in these panels is representative of two independent experiments.

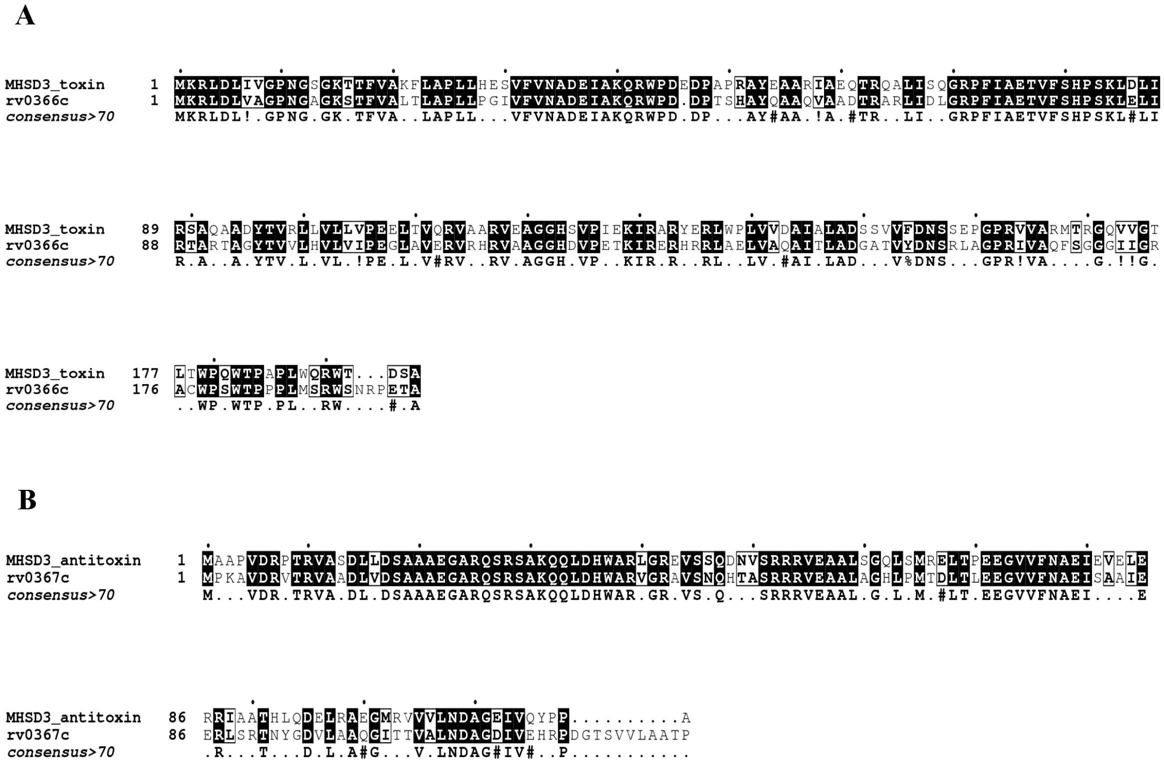

Figure S7. Alignment of PezT^{Mtb} (A) and PezA^{Mtb} (B) with MSHD3 proteins. The fully conserved residues are shown in black background.

Table S1. List of substrate-binding/active site residues in ζ_ng and AvrRxo1 toxins.

S.no	Residues in ζ_ng	Equivalent, conserved residue in $PezT^{Mtb}$ based on structure-guided alignment
1	Asp56	Asp36
2	Lys59	-
3	Asn75	-
4	His79	-
5	Ser87	-
6	Lys115	-
7	Gln135	-
8	Lys145	-
9	Arg175	Arg116
10	Arg181	His122
	**Residues in AvrRxo1**	**Equivalent conserved residue in $PezT^{Mtb}$ based on structure-guided alignment**
1	Lys166	Lys15
2	Thr167	Ser16
3	Asp193	Asp36
4	Lys196	-
5	Arg287	Arg116

Table S2: List of strains and plasmids used in the study.

**Strains**	**Description**	**References**
M. tuberculosis $H_{37}Rv$	Virulent strain of *Mycobacterium tuberculosis*	ATCC 27294
M. Smegmetis mc^2 155	Non-pathogenic fast growing mycobacteria	Kind gift from Dr. Tyagi
BL-21 (lDE3. plysS)	Expression strain for T7 promoter-based expression systems	Novagen
**Plasmids**		
pET28b	IPTG based inducible vector	Novagen
pET28b-$PezT^{Mtb}$	pET28b harboring Rv0366c from *M. tuberculosis*	This Work
pTetR	Anhydrotetracycline based mycobacterial expression episomal vector	Agarwal et al., 2018; Ehrt et al., 2005
pTetR-$PezT^{Mtb}$	pTetR harboring Rv0366c from *M. tuberculosis*	This Work
pTetR-$PezT^{MtbD36A}$	pTetR harboring Rv0366c with point mutation D36A	This Work
pTetR-$PezT^{MtbK15A}$	pTetR harboring Rv0366c with point mutation K15A	This work
pTetR-$PezT^{MtbR116A}$	pTetR harboring Rv0366c with point mutation R116A	This Work
pTetrInt	Anhydrotetracycline based mycobacterial expression integrative vector	Ehrt et al., 2005
pTetrInt-$PezT^{Mtb}$	pTetrInt harboring Rv0366c from *M. tuberculosis*	This Work
pLam12	Acetamide inducible episomal mycobacterial expression vector	Van Kessel et al., 2007
pLam12-$PezA^{Mtb}$	pLam12 harboring Rv0367c from *M. tuberculosis*	This Work

Table S3: List of primers used in the study.

	Forward(5´-3´)	Reverse(5'-3')
Primers used for construction of various overexpression strains		
pTetR-*pezT*^{Mtb}	gcatatggtgaagcggctcgatctggtcgcgc	gaagcttttacgccgtctcaggcctgttactc
pTetR-*pezT*^{MtbD36A}	cgtcaacgccgtcgaaatcgcca	tggcgatttcgacggcgttgacg
pTetR-*pezT*^{MtbR116A}	cgtcaggcatgccgtcgccgcggg	cccgcggcgacggcatgcctgacg
pTetR-pezT^{MtbK15A}	gcatatggtgaagcggctcgatctggtcgccgggcccaacggcgccggcgcgtcgacgttc	gaagcttttacgccgtctcaggcctgttactc
plam12-*pezA*^{Mtb}	gcatatggtgcccaaagccgtcgaccgcgtc	ggaattctcacggcgttggccgccagcacgac
